# Type 2 cytokines sensitize human sensory neurons to itch-associated stimuli

**DOI:** 10.3389/fnmol.2023.1258823

**Published:** 2023-10-05

**Authors:** Madison R. Mack, Yannick Miron, Fanny Chen, Paul E. Miller, Annie Zhang, Andrew Korotzer, Daniel Richman, Paul J. Bryce

**Affiliations:** ^1^Immunology and Inflammation Research Therapeutic Area, Sanofi, Cambridge, MA, United States; ^2^AnaBios, San Diego, CA, United States; ^3^Medical Affairs, Sanofi, Cambridge, MA, United States; ^4^Medical Affairs, Regeneron Pharmaceuticals, Tarrytown, NY, United States

**Keywords:** type 2 cytokines, IL-4, IL-13, IL-33, atopic dermatitis, chronic itch, sensory neurons

## Abstract

**Introduction:**

Chronic itch is a central symptom of atopic dermatitis. Cutaneous afferent neurons express receptors interleukins (IL)-4, IL-13, and IL-33, which are type 2 cytokines that are elevated in atopic dermatitis. These neuronal cytokine receptors were found to be required in several murine models of itch. Prior exposure of neurons to either IL-4 or IL-33 increased their response to subsequent chemical pruritogens in mice but has not been previously examined in humans. The objective of the present study was to determine if type 2 cytokine stimulation sensitizes sensory neurons to future itch stimuli in a fully human *ex vivo* system.

**Methods:**

We measured calcium flux from human dorsal root ganglia cultures from cadaveric donors in response to pruritogens following transient exposure to type 2 cytokines. We also measured their effect on neuronal calcium flux and changes in gene expression by RNA sequencing.

**Results:**

Type 2 cytokines (IL-4, IL-13, and IL-33) were capable of sensitizing human dorsal root ganglia neurons to both histaminergic and nonhistaminergic itch stimuli. Sensitization was observed after only 2 h of pruritogen incubation. We observed rapid neuronal calcium flux in a small subset of neurons directly in response to IL-4 and to IL-13, which was dependent on the presence of extracellular calcium. IL-4 and IL-13 induced a common signature of upregulated genes after 24 h of exposure that was unique from IL-33 and non-type 2 inflammatory stimuli.

**Discussion:**

This study provides evidence of peripheral neuron sensitization by type 2 cytokines as well as broad transcriptomic effects in human sensory ganglia. These studies identify both unique and overlapping roles of these cytokines in sensory neurons.

## Introduction

Chronic itching is the most common symptom in patients with atopic dermatitis and has a significant negative impact on patients’ quality of life (Ständer et al., 2019). The molecular mechanisms of itch have been the focus of extensive research over the past 10 years and a role for immune mediators such as cytokines and chemokines has recently emerged (Du et al., 2022). The type 2 cytokines interleukins (IL)-4, IL-13, and IL-33 are central in the inflammatory pathogenesis of atopic dermatitis (Langan et al., 2020) and implicated to play an additional role in itch sensation in murine models. The shared receptor subunit for IL-4 and IL-13, IL-4Rα, is expressed in mouse and human sensory neurons, and murine studies implicate this receptor specifically in transmitting itch sensation (Oetjen et al., 2017; Campion et al., 2019). Similarly, the IL-33 receptor subunit *IL1RL1* (ST2) is also expressed by dorsal root ganglion (DRG) neurons and was found to mediate itch associated with contact dermatitis and xerosis in mice (Liu et al., 2015; Trier et al., 2022). However, how cytokines modulate neuronal activity and whether this biology is shared between rodents and humans remains unknown.

Chemical itch is frequently categorized into histaminergic and nonhistaminergic signaling pathways. In humans, histaminergic itch signaling is largely involves H1R expressed by primary sensory neurons (Guo et al., 2022). H1R is coupled with Gq/11 protein to induce activation of phospholipase Cβ3, leading to increase of intracellular Ca^2+^ in DRG neurons via nonselective cation channel Transient Potential Vanilloid family member V1 (TRPV1). In contrast, histamine-independent signaling occurs through receptors such as MAS-related G-protein-coupled receptor X1 (MRGPRX1 in human, in mice MrgprC11), which binds a variety of itch-inducing ligands including chloroquine and the proenkephalin-derived peptide BAM8-22 (Liu et al., 2009; Sikand et al., 2011). MRGPR-mediated itch utilizes the ion channel TRPA1 for downstream signal transduction (Wilson et al., 2011). The direct pruritogenic activity of these pathways have been shown by observing scratching behaviors in mice (Ru et al., 2017) and reported itch sensation in humans (Li et al., 2021) following subcutaneous injection.

How type 2 cytokines fit into this paradigm is unclear. Initial studies in mice indicate that injection of IL-4, IL-13, or IL-33 did not provoke rapid scratching behaviors (Oetjen et al., 2017; Trier et al., 2022), unlike histamine or another cytokine, IL-31 (Cevikbas et al., 2014). However, a later study did demonstrate that IL-4 and IL-13 can induce acute scratching behavior in mice (Campion et al., 2019). An alternative hypothesis to explain the impact of these cytokines receptors on chronic itch emerged after Oetjen et al. and Trier et al. concluded that prior exposure to either IL-4 or IL-33 (respectively) altered the responsiveness of mouse neurons to subsequent pruritogenic ligands, such as histamine and chloroquine (BAM8-22) (Oetjen et al., 2017; Campion et al., 2019; Trier et al., 2022). In neuronal cell cultures, exposure to IL-4 or IL-33 primed neurons to respond to subthreshold concentrations of canonical itch ligands, suggesting that type 2 cytokines sensitize pruriceptors to subsequent stimuli. However, in part due to the challenges in obtaining human neural tissues, the relevance of these findings to human itch sensation has remained uncertain.

To better understand the mechanisms underlying this phenomenon, we tested human sensory neurons obtained from cadaveric DRG for direct responses to type 2 cytokines IL-4, IL-13, and IL-33 by visualizing intracellular calcium. Further, we tested sensitization by exposing neurons to the cytokines for 2 h prior to histamine and BAM8-22. In addition, we also measured whole-transcriptome changes in human DRG cultures after 24 h of cytokine exposure. Our findings here support the importance of type 2 cytokines in priming neurons for subsequent stimulation by pruritogens and establishes the gene expression changes occurring upon neuronal exposures to these cytokines.

## Materials and methods

All experiments were conducted on isolated human DRG (hDRG) neurons from cadaveric organ donors. All human tissues used for the study were obtained by legal consent from organ donors in the United States. Donor demographics can be found in Supplementary Table S1.

### Human dorsal root ganglia culture

Human DRGs were transferred into a dissection vessel containing a cold (4°C) proprietary dissection solution. DRGs were maintained completely submerged during dissection to remove connective tissue from the ganglia. hDRG was enzymatically dissociated as per AnaBios’ proprietary methodologies. Dissociated cells were seeded on 96-well plastic bottom plates (Corning) that had been pre-coated with poly-D-lysine. Cells were maintained in culture at 37°C with 5% CO_2_ in 200 μL DMEM/F12 supplemented with 10% horse serum (Thermo Fisher Scientific, Waltham, Massachusetts), 25 ng/mL hNGF (Cell Signaling Technology, Danvers, MA), 25 ng/mL GDNF (PeproTech, London, UK), Gem21 NeuroPlexand (Gemini Bio, West Sacramento, California) penicillin/streptomycin (Thermo Fisher Scientific, Waltham, Massachusetts). Half of the culture media was replaced with fresh media every 3 days.

### Calcium imaging

Cells were loaded with the fluorescent calcium indicator, 3 μM Fluo-8 AM (AAT Bioquest, Pleasanton, CA) in Calcium Imaging Buffer, for 30 min at room temperature in the dark. Intracellular calcium transients were measured by changes in fluorescence intensity of the Fluo-8-calcium indicator upon excitation at 480 nm and the signal emitted at 520 nm was recorded using a pcoEDGE sCMOS camera mounted on an IX71 inverted microscope (Olympus, Waltham, MA, United States). Imaging sampling rate was 0.2 Hz in time lapse mode.

### Neuronal potentiation by type 2 cytokines and chemical pruritogens

Cultures were stimulated with 500 nM IL-4, IL-13, IL-33 (R&D Systems, Minneapolis MN) or the combination of IL-4 and IL-13 for 2 h. A cocktail of inflammatory mediators—1 μM histamine (Sigma-Aldrich, St-Louis, MO), 1 μM serotonin (Sigma-Aldrich, St-Louis, MO), 100 nM bradykinin (Sigma-Aldrich, St-Louis, MO), and 1 μM prostaglandin E_2_ (Tocris Bioscience, Bristol, UK)—previously used to demonstrate peripheral sensitization in this system (Castro et al., 2019) was used as a Control Inflammatory Stimuli (CIS). Following cytokine exposure, cells were washed and then challenged with pruritogens BAM8-22 (2 μM; Tocris Bioscience, Bristol, UK) or histamine (10 μM) or TRP agonists Allyl isothiocyanate (AITC; 25 μM; Sigma-Aldrich, St. Louis, MO) or capsaicin (100 nM; Sigma-Aldrich, St. Louis, MO) for 30 s. Cells were then washed a second time and stimulated with TRPV1 agonist capsaicin (200 nM) as an assay control by direct application for 20 s.

### RNA isolation and RNA sequencing

After removing culture medium, dissociated DRG cultured neurons were immersed in RNA*later* (Thermo Fisher Scientific, Waltham, MA, United States), scraped off the plate and stored in Eppendorf Tubes at 0°C. After spinning down RNA*later* tubes with 3,000 x g for 5 min, RNA*later* was removed and resuspend in 350 μL RLT buffer containing 2-mercaptoethanol (Acros Organics, Fair Lawn, NJ, United States). Samples were mixed thoroughly by shaking for 15–30 s. Total RNA was extracted using the RNeasy Mini Kit (QIAGEN, Valencia, CA, United States) and quantified using the Nanodrop ND-1000 (Thermo Fischer Scientific, Waltham, MA, United States). The extracted total RNA was used to assemble Illumina platform libraries and sequenced on a NovaSeq 6000. Raw and processed data are available at GEO archive GSE242488.

### Analysis of single cell RNA sequencing

Raw data from Nguyen et al. (2021) were reanalyzed and visualized with 2D t-distributed stochastic neighbor embedding (t-SNE) with Louvain clustering (resolution = 1) in BioTuring Talk2Data browser. The Louvain algorithm clusters cells through modularity function maximization and determines the number of clusters automatically (Liu, 2021). Data visualization was performed using Vinci (BioTuring, San Diego, CA, United States).

### Statistical analysis

Calcium imaging data analysis was conducted in MetaMorph7.8.3. Statistical analyses were conducted in GraphPad Prism for *in vitro* assays. Transcriptomic differential gene expression analysis was conducted in r using DESeq2 (Love et al., 2014). The complete results of differential gene expression analysis are available in Supplementary Table S2.

## Results

### Type 2 cytokines potentiate calcium flux in response to pruritic stimuli

**Figure 1:**
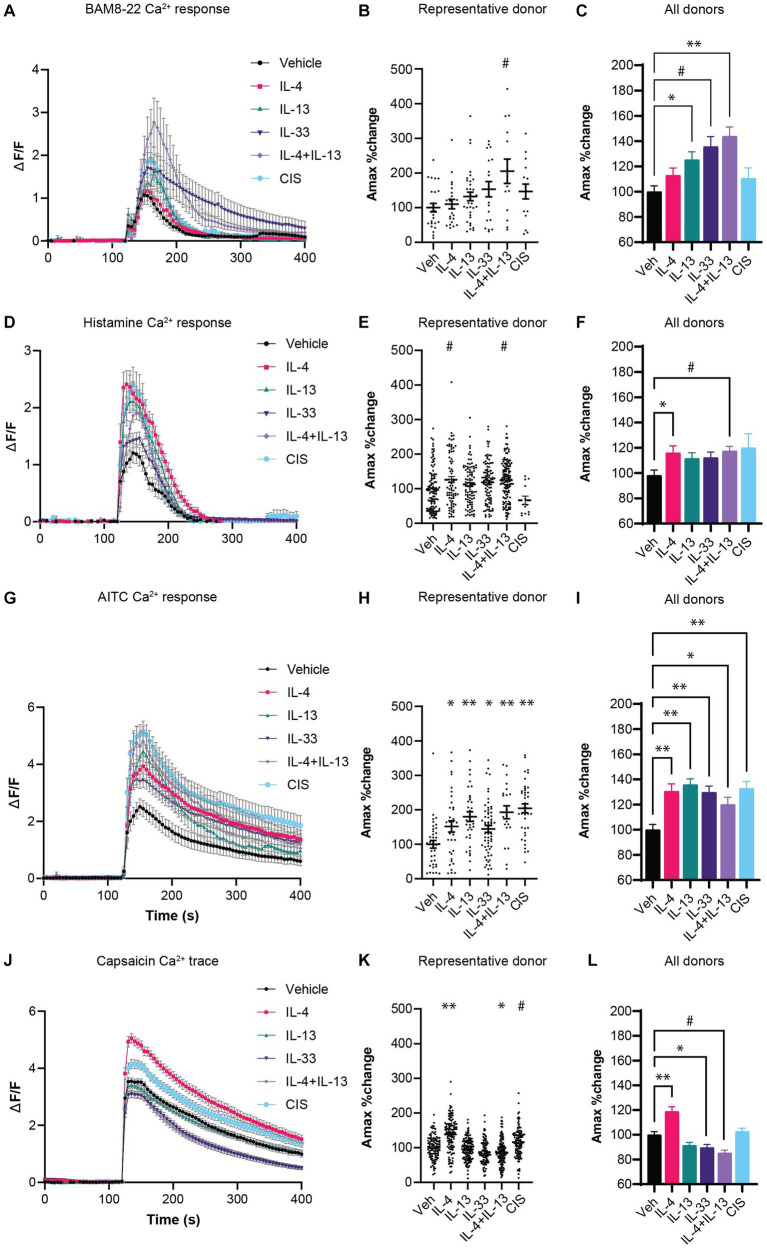
**(A)** Calcium response of neurons responding to non-histaminergic agonist BAM8-22 (2 μM). **(B)** A_max_ of individual neurons responding to BAM8-22 from a representative donor. **(C)** Average A_max_ of neurons responding to BAM8-22 normalized to the average vehicle-control condition for the respective donor (*N* =5 donors). **(D)** Calcium response of neurons responding to histamine (10 μM). **(E)** Peak Ca^2+^ flux amplitude (A_max_) of individual neurons responding to histamine from a representative donor. **(F)** Average A_max_ of neurons responding to histamine normalized to the average vehicle-control condition for the respective donor (*N* = 4 donors). **(G)** Calcium response of neurons to TRPA1 agonist AITC (25 μM). **(H)** Peak Ca^2+^ flux amplitude (A_max_) of individual neurons responding to AITC from a representative donor. **(I)** Average A_max_ of neurons responding to AITC normalized to the average vehicle-control condition for the respective donor (*N* = 3 donors). **(J)** Calcium response of neurons to TRPV1 agonist capsaicin (100 nM). **(K)** A_max_ of individual neurons responding to capsaicin from a representative donor. **(L)** Average A_max_ of neurons responding to capsaicin normalized to the average vehicle-control condition for the respective donor (*N* = 3 donors). CIS: Bradykinin (100 nM), PGE_2_ (1 μM) serotonin (1 μM), histamine (1 μM); Veh: Vehicle; *p < 0.05, ^#^*p* < 0.01, **p < 0.001, by One-Way ANOVA with Dunnett correction for multiple comparisons. AITC, Allyl isothiocyanate; CIS, control inflammatory stimuli; hDRG, human dorsal root ganglion; IL, interleukin; TRP, transient receptor potential.

We performed calcium imaging of hDRG cultures from human cadaveric donors primed with different type 2 cytokines to test whether prior exposure could sensitize neurons to subsequent chemical pruritogens BAM8-22 and histamine. The frequency of neurons responding to BAM8-22 in vehicle conditions varied by donor, from 6 to 38% of DRG neurons, and no change in the frequency of responding neurons was observed following cytokine sensitization (Supplementary Figure S1A). However, following sensitization, we observed a significant increase in the amplitude of intracellular calcium flux in neurons exposed to IL-13, IL-33, and most robustly to a combination of IL-4 and IL-13, compared to vehicle controls (Figures 1A–C). IL-4 and the control inflammatory stimuli (CIS) showed a trend toward increased calcium flux to BAM8-22 compared to vehicle controls, but the effect was not statistically significant (Figure 1C).

For histamine, 22–44% of neurons responded to histamine stimulation at baseline. Under the same sensitization conditions, no significant change in the frequency of responding neurons occurred except for a decreased response rate in CIS-exposed neurons (Supplementary Figure S1B). Since the CIS treatment includes histamine, this desensitization effect is expected (Shim et al., 2007). However, similar to the BAM8-22 results, a significant increase in the amplitude of calcium flux was observed in cultures pre-treated with IL-4 and IL-4 + IL-13 compared to the vehicle controls (Figures 1D–F). We observed a trending but not significant increase in calcium flux in response to IL-13, IL-33, and CIS pre-treatment (Figure 1F).

Transient receptor potential (TRP) channels are thought to be required for the rapid calcium flux induced by cytokine receptors on neurons by coupling non-ion channel receptors to the cation channels TRPA1 and/or TRPV1 to transduce action potential in itch-sensory neurons (Feng et al., 2017; Wilzopolski et al., 2021). These channels can be sensitized by inflammatory stimuli to lower their activation threshold, which may increase the responsiveness of neurons to associated pruritogenic stimuli (Gouin et al., 2017). Therefore, we tested whether cytokine exposure sensitized TRPA1 and TRPV1 using chemical ligands AITC and capsaicin, respectively. Two-hour exposure to all type 2 cytokine conditions resulted in a dramatic increase the AITC-induced Ca^2+^ A_max_ (Figures 1G–I), demonstrating robust sensitization of TRPA1 channel. In contrast, we did not observe this level of sensitization to the TRPV1 ligand capsaicin. We observed sensitization in response to IL-4 in one donor, while exposure to IL-33 and IL-4 + IL-13 resulted in a slight repression of capsaicin-induced Ca^2+^ A_max_ (Figures 1J–l). Like the pruritogens BAM8-22 and histamine, we did not observe any changes in the percent of neurons responding to AITC and capsaicin following cytokine pre-treatment (Supplementary Figures S1C,D).

Overall, these data demonstrate that IL-4, IL-13 as well as IL-33 can increase the amplitude of intracellular calcium flux in response to histaminergic and non-histaminergic ligands as well as sensitize the cation channel TRPA1.

### Expression of type 2 cytokine receptors on human DRG neurons with single-cell resolution

Various studies in mice have demonstrated a role for neuronal IL-4Rα and the IL-33R ST2 in itch sensation, although additional cell types in the DRG express these receptors (Thakur et al., 2014; Avraham et al., 2022; Trier et al., 2022). To understand whether this translates to human itch-sensory neurons, we reanalyzed a recently published single nuclei RNA-seq data set from human DRG neurons for expression of type 2 cytokine receptors and signaling components (Nguyen et al., 2021). The authors experimentally and computationally isolated neurons from bulk DRG and identified a range of diverse transcriptomic classes of human somatosensory neurons.

**Figure 2:**
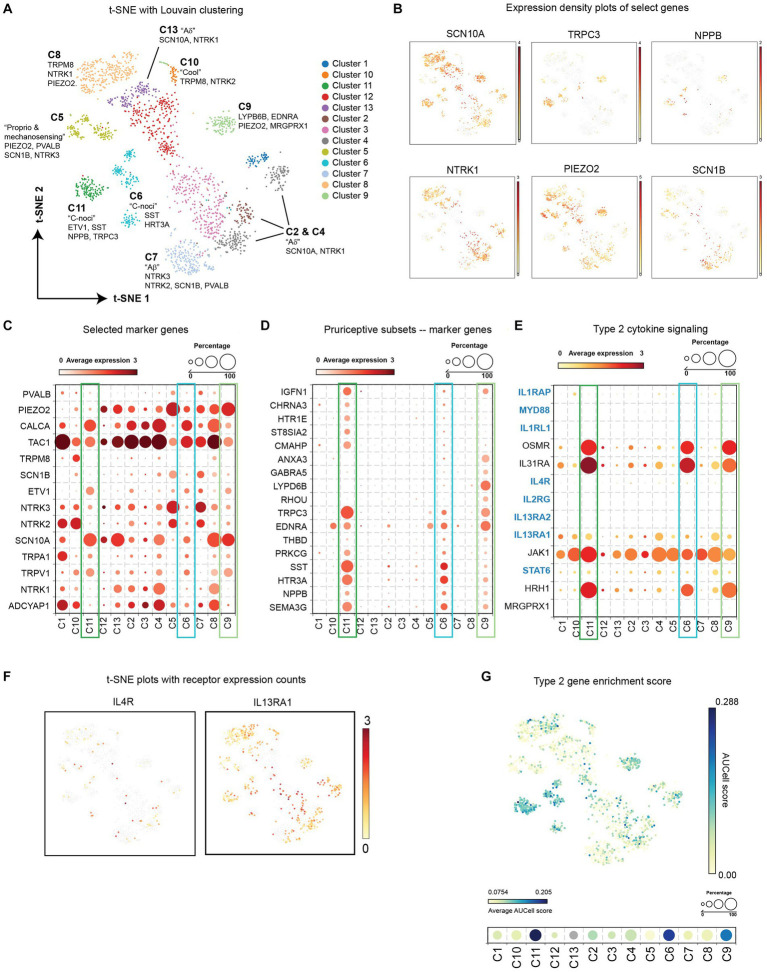
**(A)** t-SNE with Louvain clustering of DRG neurons from reanalyzed single nuclei RNA sequencing (Nguyen et al., 2021). **(B)** Expression density of select genes on the t-SNE plot. **(C)** Average expression level and frequency of selected marker genes of established neuronal subsets within each cluster. **(D)** Average expression level and frequency of genes selected during marker. Analysis of putative pruriceptors clusters C6, C9, and C11 from BioTuring “*find markers*” function. **(E)** Average expression level and frequency of select genes involved in type 2 cytokine receptor signaling within each cluster. **(F)** t-SNE plots depicting the expression density of type 2 cytokine receptors IL-4Rα and IL-13Rα1. **(G)** Enrichment scores of type 2 cytokine-associated signaling genes highlighted in blue in panel **(E)**. AUCell score for each neuron is represented in the indicated color scale for each cell in the scatter plot (top) and as an average for each cluster (bottom). DRG, dorsal root ganglion; IL, interleukin; t-SNE, t-distributed stochastic neighbor embedding.

Raw data from 1,837 neuronal nuclei were reanalyzed in BioTuring Talk2Data browser and visualized with t-SNE. Louvain clustering identified 13 populations (Figure 2A), which correlated well with the 14 populations initially identified by Nguyen et al. In this clustering method, putative pruriceptive populations C6, C9, and C11 were identified based on marker expression such as *NPPB*, *SST*, and *SCN10A,* and overlap with previous pruriceptor designations H10 and H11 by Nguyen et al. (Figures 2B,C). Marker gene analysis from BioTuring Talk2Data of these three populations confirmed selective expression of *NPPB* and *SST* as well as additional itch-associated receptors such as *TRPC3* (Liu et al., 2021) and *HTR3A* (Tian et al., 2016) (Figure 2D). These cells were also strongly enriched for expression of histamine receptor *HRH1*, and IL-31 and Oncostatin M receptor signaling subunits *IL31RA* and *OSMR* (Tseng and Hoon, 2021) (Figure 2E). Interestingly, the cells in cluster C6 are a mixture of cells previously attributed to H10/C9 and H11/C11, which may indicate an intermediate or additional pruriceptive subpopulation.

In all three pruriceptive subpopulations, we detected a small number of neurons expressing *IL4R* and a larger subset expressing *IL13RA1* (Figures 2E,F). No *IL1RL1* (ST2) transcripts were detected in any neuronal nuclei (Figure 2E); however, it is important to note that the depth of sequencing on this platform limits detection of less-abundant transcripts. Therefore, we performed an additional analysis looking at the composite enrichment of receptors and signaling components associated with IL-4, IL-13, and IL-33 using the AUCell score (Aibar et al., 2017) implemented in BioTuring’s Talk2Data browser (genes used are highlighted in blue in Figure 2E). Surprisingly, the cells with the largest AUCell score were concentrated in putative pruriceptive clusters C6, C9, and C11, suggesting that these subsets may be especially attuned to type 2 cytokine signaling (Figure 2G).

### Direct activation of neuronal calcium flux by type 2 cytokines

**Figure 3:**
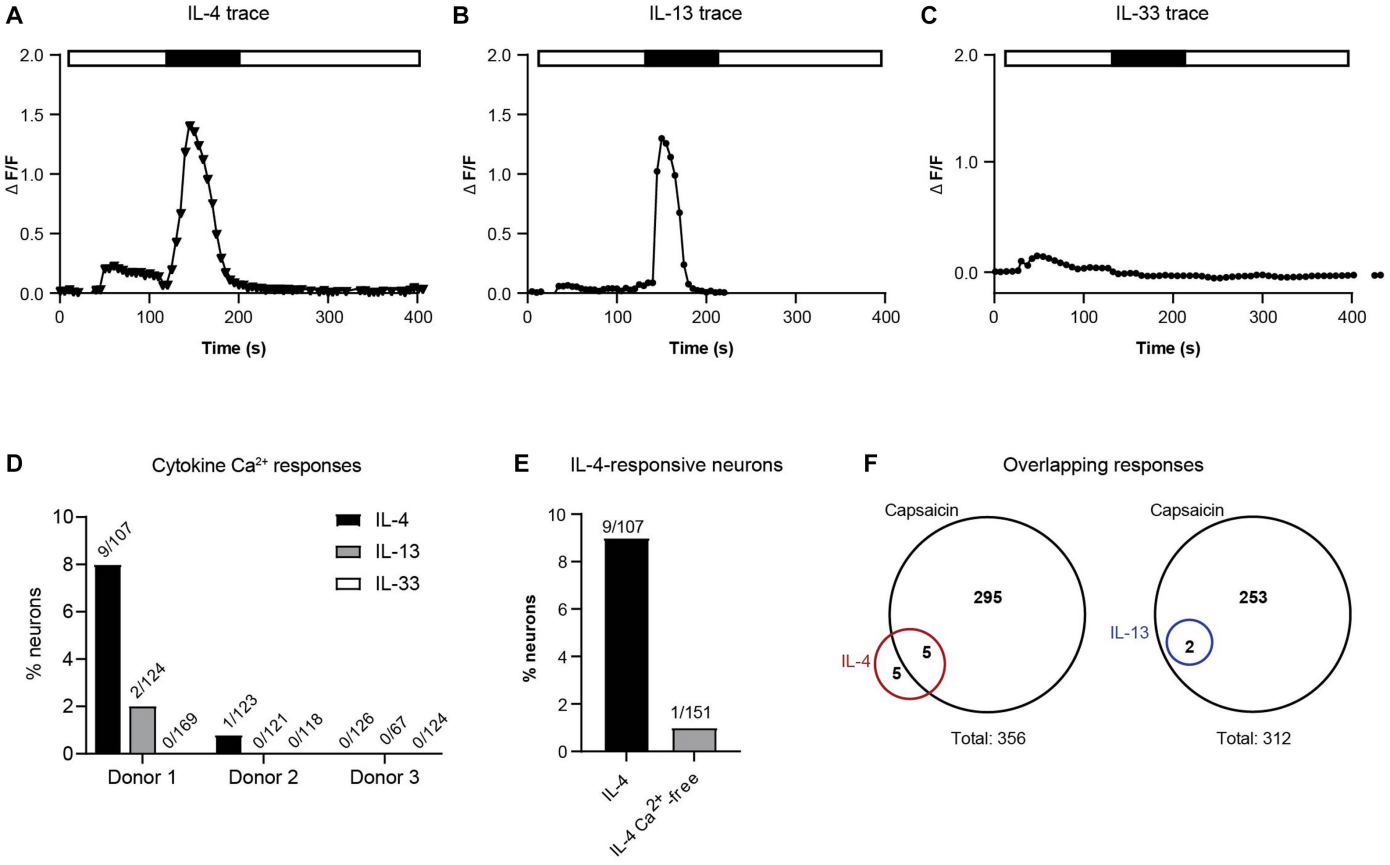
**(A)** Intracellular calcium flux of a representative neuron responding to IL-4. **(B)** Intracellular calcium flux of a representative donor neuron responding to IL-13. **(C)** Intracellular calcium flux of a representative neuron responding to IL-33. **(D)** Percent of responding neurons to each cytokine by donor (number of response/total neurons). **(E)** Neurons responding to IL-4 with and without extracellular calcium. **(F)** Fraction of IL-4- and IL-13-responsive neurons that also respond to TRPV1 agonist capsaicin (number of responsive neurons; 3 donors). IL, interleukin.

To test the hypothesis that these cytokines (IL-4, IL-13, and IL-33) can act directly on human sensory neurons, we measured intracellular calcium flux in response to direct application of IL-4, IL-13, and IL-33 on hDRG cultures. Acute calcium flux was observed in a small subset of neurons in response to both IL-4 and IL-13 stimulation (Figures 3A,B). Ten out of 356 neurons (2.8%) across three individual donors responded to IL-4 (Figures 3A,D) and two out of 312 neurons (0.64%) across three individual donors responded to IL-13 (Figures 3B,D). Although these responses were rare, the degree and duration of calcium flux was similar in magnitude and duration to the baseline levels of histamine and BAM8-22 (Figures 3A,D). We did not detect any neuronal calcium response to IL-33 in any of the donors (411 total neurons) (Figure 3C).

Calcium flux downstream of cytokine receptors is thought to occur through cooperative activity of ion channels to permit influx of extracellular calcium into the cytoplasm. To determine whether type 2 cytokines were stimulating this type of calcium response, we repeated exposure of neurons to IL-4 in the absence of extracellular calcium. In this experiment, removal of extracellular calcium decreased the number of responsive neurons by 90% (Figure 3E). We also found that 50% of IL-4-responsive neurons and 100% of the IL-13-responsive neurons were also responsive to the TRPV1 agonist capsaicin (Figure 3F). Taken together, these data suggest that similar to findings in mice (Oetjen et al., 2017; Trier et al., 2022), human DRG neurons utilize ion channels to induce extracellular calcium flux after type 2 cytokine priming.

### Overlapping and unique gene expression changes in response to type 2 cytokines

**Figure 4:**
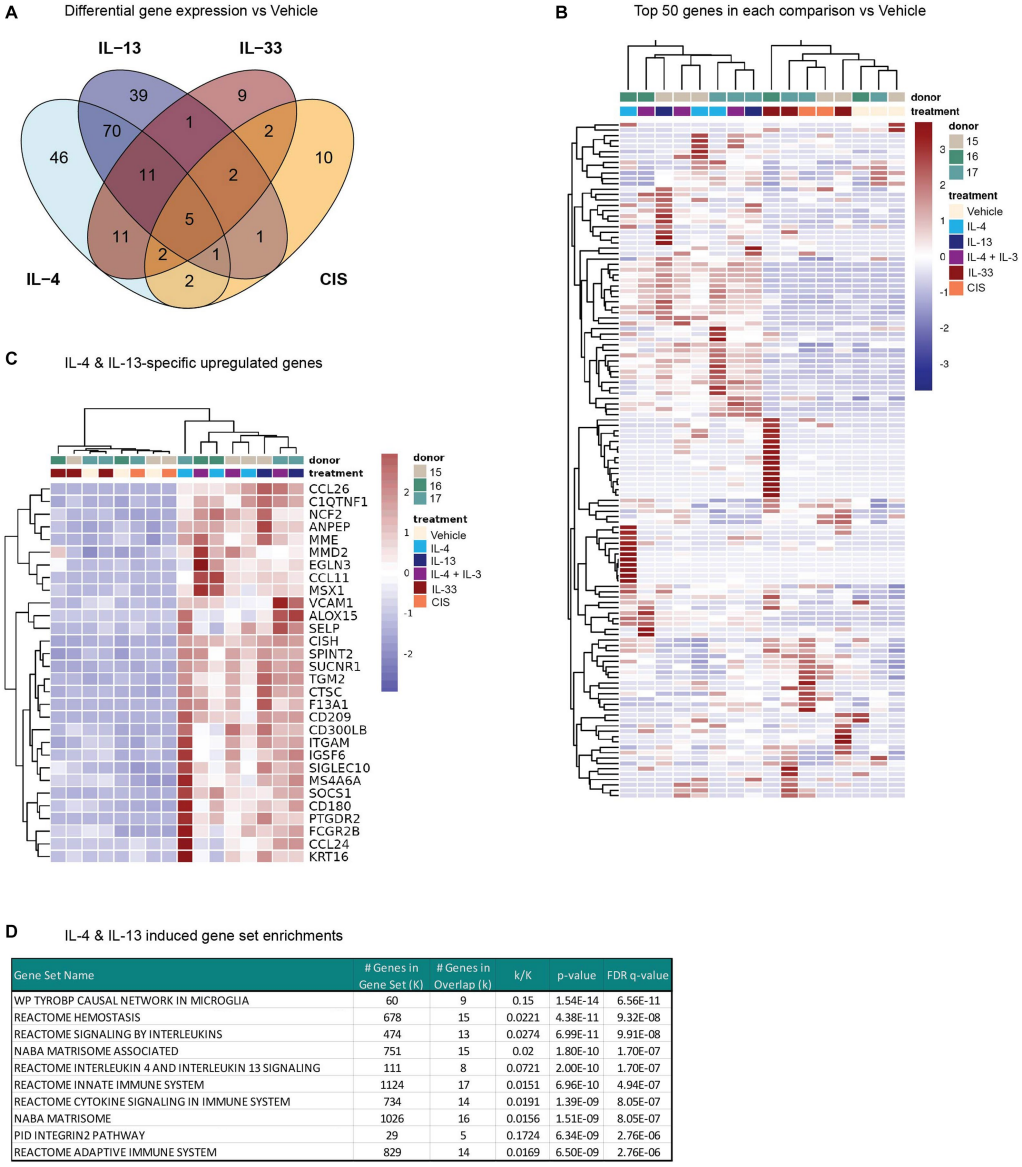
Whole-transcriptome changes in human DRG cultures after 24 h of cytokine exposure. **(A)** Venn diagram indicating the number of differentially expressed genes for each treatment vs. Vehicle (*Padj* < 0.05). **(B)** Heatmap of the expression levels (normalized FPKM) of the top 50 genes by log2 fold change in each comparison vs. Vehicle for each donor. **(C)** Heatmap depicting the expression levels of a subset of genes that were selectively upregulated (log2 fold change >1) by either IL-4 or IL-13 treatment, but not IL-33- or CIS-treatment. **(D)** Top 10 IL-4 & IL-13 induced gene set enrichment analysis results. CIS, control inflammatory stimuli; DRG, dorsal root ganglion; IL, interleukin.

In addition to the rapid changes in calcium flux following prior cytokine exposure, we also measured whole-transcriptome changes in human DRG cultures after cytokine exposure. Since the precise timing of gene expression changes in response to JAK-STAT or MyD88 signaling in human sensory neurons is unknown, we chose a time point of 24 h to fully capture the transcriptome-wide changes to type 2 cytokines. Total RNA from DRG cultures incubated with vehicle or cytokines was isolated from three donors per treatment and sequenced to determine global gene expression changes. We were unable to sequence samples from two of the conditions due to insufficient material from these donors. Despite this, we found that each cytokine treatment induced the expression of a unique and overlapping set of genes in hDRG, with the most overlap found between IL-4 and IL-13 (Figure 4A). Responses to IL-33 and CIS greatly varied between donors (Figure 4B), yielding few significantly induced genes.

Of the protein-coding differentially expressed genes, the majority of IL-13-induced genes were shared with IL-4 (Figure 4A). Both IL-4 and IL-13 enhanced the expression of several genes in our human DRG cultures previously identified as IL-13–responsive (Miron et al., 2022), such as *NPPB, TGM2*, *CTSC*, and *SUCNR1*, as well as *CCL11*, *CCL26*, and *ALOX15.* Hierarchical clustering of samples by the top differentially expressed genes across all conditions shows clear grouping of IL-4/13-treatment samples with each other and separate from IL-33 and CIS (Figure 4B). We identified 70 genes unique and shared across IL-4 and IL-13 treatments, but not IL-33 or CIS (Figures 4A,C). Gene set enrichment analysis (Subramanian et al., 2005) of this subset identified significant overlap in MSigDB (Liberzon et al., 2011) gene sets associated with interleukin signaling, including IL-4 and IL-13 signaling, as well as with extracellular matrix (matrisome) (Figure 4D). These data indicate that IL-4 and IL-13 have overlapping effects on gene expression in the DRG, which is largely conserved with genes induced by IL-4 and IL-13 in other tissues.

## Discussion

Despite recent advancements in our understanding of the cellular and molecular mechanisms of itch, human pruriceptive sensory neurons remain poorly understood. Substantial challenges in obtaining living human neural tissues as well as determining causal relationships in trials involving human subjects limit their study. While rodents and primates share many neuronal signaling pathways, the discrete functional populations are not fully conserved and, unlike with murine studies, only a few studies have described human and primate sensory neurons with single cell resolution (Nguyen et al., 2021; Tavares-Ferreira et al., 2021; Jung et al., 2023). For example, Nguyen et al. identified that populations of pruriceptive neurons in humans had no clear transcriptional correlation in mice and expressed polymodal receptor functionality (Nguyen et al., 2022). In the present study, we found that these populations of DRG neurons express type 2 cytokine receptors *IL4RA* and *IL13RA1* at the single cell level. These results are consistent with the detected low-level expression of *IL13RA1* and *IL4RA* transcripts in sorted murine NPPB-tdTomato^+^ neurons (Tseng and Hoon, 2021) as well as co-expression in presumptive itch-sensory subsets (NP1-3) from single-cell studies in mice (Chiu et al., 2014; Usoskin et al., 2015). While no transcripts for IL-33 receptor ST2 (*IL1RL1*) or Type 1 IL-4 receptor subunit *IL2RG* were detected in these subsets, the low depth of sequencing possible in this study limits conclusions about negative findings.

Results from this *ex vivo* study build on the recent findings by Miron et al. in several ways (Miron et al., 2022). First, we established that IL-4, in addition to IL-13, can potentiate calcium responses to histaminergic and non-histaminergic stimuli in human sensory neurons. Miron et al. found similar sensitization results to histamine and BAM8-22, as well as serotonin, after 20 min as well as 24 h of IL-13 exposure. These data are supported by previous findings that IL-4 can rapidly modulate calcium homeostasis in the context of cholinergic signaling in human and bovine smooth muscle cells (Madison and Ethier, 2001) and IL-13 can induce β-adrenergic hypo-responsiveness (Laporte et al., 2001). Although these cytokines share a common signaling cascade, they each have different binding kinetics and unique co-receptor complexes that can result in unique biology (Junttila, 2018). These differences are important to understand in the context of targeted biologic therapies against IL-4 and IL-13 used in the setting of chronic type 2 inflammation and pruritic disorders. Second, we also report that IL-33 can similarly sensitize neuronal calcium responses, demonstrating that the biology of type 2 cytokine sensitization extends beyond IL-4Rα signaling. However, the mechanism connecting type 2 cytokine exposure to enhanced calcium flux in response to subsequent stimuli in human DRG neurons remains unexplored.

One hypothesis is that type 2 cytokine signaling modulates TRPV1 and TRPA1 ion channels, which have been shown in mice to be critical for coupling G-protein couple receptor signaling such as MRGPRX1 and histamine receptors to an influx of extracellular Ca^2+^. In the present study we found that exposure to type 2 cytokines resulted in a notable increase the AITC-induced Ca^2+^, providing the first evidence of TRPA1 channel potentiation by type 2 cytokines. How this sensitization occurs remains unclear. One study in rodents demonstrated that purinergic G-protein couple receptor P2RX_3_ promoted hyperalgesia in trigeminal ganglia via phosphorylation of serine residues on TRPV1 (Salomon et al., 2013). Indeed, TRP channel phosphorylation and modulation of TRP channel insertion into the plasma membrane can promote hyperresponsivity to stimuli (Meng et al., 2021). Such a mechanism may explain how type 2 cytokines induced the influx extracellular calcium and potentiate neuronal responses to multiple pruritogenic stimuli.

We did not observe a similar sensitization of TRPV1 in this study. Although some sensitization following IL-4 treatment was observed, a slight repression of capsaicin responses occurred following IL-33 and IL-4 + IL-13 exposure. This differential effect on TRPA1 vs. TRPV1 may be due to differences in the mechanisms of desensitization of these channels (Akopian et al., 2007; Moparthi et al., 2020). For example, TRPA1 can be activated rather than repressed at certain intracellular Ca^2+^ concentrations (Jordt et al., 2004). However, future work is needed to fully interrogate the mechanisms underlying TRP channel modulation by type 2 cytokine exposure.

In addition to localized membrane signaling, chronic exposure to type 2 cytokines also has the capacity to induce transcription-dependent changes relevant to chronic itching, such as upregulation of pruriceptors or changes in neuronal growth or branching. We measured whole-transcriptome changes in human DRG cultures stimulated with IL-4, IL-13, and IL-33 for 24 h. RNA-Seq analysis demonstrated changes in expression of a considerable number of genes, including 70 genes that were uniquely upregulated >2-fold by either IL-4 or IL-13. Our finding is consistent with recently published data reporting an increase in transcripts related to atopic dermatitis, inflammation, and itch after IL-13 stimulation in human DRG cultures (Miron et al., 2022). In our study, IL-4 treatment induced a highly overlapping set of genes to IL-13. We did not detect any upregulation of receptors of tested ligands in this study; however, it should be noted that transcriptomic changes were observed after 24 h, while functional sensitization was measured after 2 h of cytokine exposure.

It also is important to consider that the transcriptomic experiments are conducted on bulk RNA from a mixed culture of cell types isolated from human DRGs. This includes neurons as well as a variety of glia, stromal cells, and immune cells (Valtcheva et al., 2016; Ray et al., 2018; Avraham et al., 2022). Such non-neuronal cells may play an important role in modulating itch sensation. Increased expression of glial fibrillary acidic protein, a marker of astrocyte activation, has been observed in the spinal cords of mice undergoing different chronic itch disease models (Shiratori-Hayashi and Tsuda, 2020, 2021) and the IL-33 receptor ST2 is expressed on spinal dorsal horn astrocytes and contributes to both STAT3-dependent astrocytic activation and chronic itch (Du et al., 2019). Whether spinal glia regulate the phenomenon of itch sensitization in response to IL-4 or IL-13 remains to be tested.

Our study has several important limitations. First, our study is limited in scope to a single dose and time point for each cytokine and agonist. Unlike studies in research animals, acquisition of human sensory neurons is more limited and required a restricted scope of work. In addition, we observed a significant amount of variability in responses between donors. While it would be interesting to understand whether this variability relates to the donors’ exposures, genetics, and other factors, these details are not available for our de-identified donor population. Although we are unable to draw conclusions about negative findings due to these limitations, it is important to continue to interrogate human primary cells whenever possible to translate the detailed work done in model systems to humans and hopefully 1 day to patient therapies.

Taken together, our data indicate that type 2 cytokines IL-4, IL-13, and IL-33 have acute and chronic effects on neuronal excitation, pruritogen sensitization, and transcript regulation in human sensory neurons. In this study, we demonstrate that IL-4 and IL-13 have overlapping functions in neuronal sensitization and gene expression changes in human DRG. Furthermore, IL-33 also promoted enhanced responsiveness, indicating that type 2 cytokine impact on neuronal sensitization extends beyond IL-4Rα and JAK–STAT signaling. The mechanism by which IL-4, IL-13, and IL-33, can sensitize human DRG neurons to subsequent stimuli, as well as how this impacts itch sensation in chronic inflammatory diseases such as AD, remains an exciting area for future investigation.

## Data availability statement

The data presented in this study are deposited in the Gene Expression Omnibus (GEO) repository. This data can be found here: https://www.ncbi.nlm.nih.gov/geo/query/acc.cgi?acc=GSE242488 accession number GSE242488.

## Ethics statement

Ethical approval was not required for the studies involving humans because studies involved tissue donated by organ donors for research purposes. Legal consent was provided by the organ donor or next of kin according to the policies established by the United Network for Organ Sharing (UNOS). The studies were conducted in accordance with the local legislation and institutional requirements. The human samples used in this study were acquired from AnaBios Corporation’s procurement network, which includes only US-based Organ Procurement Organizations and Hospitals. Written informed consent to participate in this study was not required from the participants or the participants’ legal guardians/next of kin in accordance with the national legislation and the institutional requirements.

## Author contributions

MM: Conceptualization, Investigation, Writing – review & editing, Data curation, Formal analysis, Writing – original draft, Visualization. YM: Investigation, Methodology, Writing – review & editing, Data curation, Formal analysis, Supervision. FC: Writing – review & editing, Investigation, Methodology. PM: Writing – review & editing, Methodology, Project administration, Resources. AZ: Writing – review & editing, Supervision. AK: Writing – review & editing, Supervision. DR: Writing – review & editing, Conceptualization, Funding acquisition, Project administration, Supervision. PB: Writing – review & editing, Conceptualization, Supervision.

## Abbreviations

AITC: Allyl isothiocyanate
IL: Interleukin
CIS: Control inflammatory stimuli
CVA: Cerebrovascular accident
DRG: Dorsal root ganglion
GSW: Gunshot wound
ICH: Intracerebral hemorrhage
hDRG: Human dorsal root ganglion
OSM: Oncostatin M
TRP: Transient receptor potential
t-SNE: t-distributed stochastic neighbor embedding
