# Supplementary material for: Type 2 cytokines sensitize human sensory neurons to itch-associated stimuli
Source: Front Mol Neurosci. 2023 Oct 5;16:1258823. doi: 10.3389/fnmol.2023.1258823 (PMC10586051; doi:10.3389/fnmol.2023.1258823)
Supplement: Supplementary file 1 [file Table_1.docx]

Type 2 Cytokines Sensitize Human Sensory Neurons to Itch-associated Stimuli

Supplementary Table 1. Donor demographics

|  | Age | Sex | BMI | Ethnicity | Cause of death | Study treatment |
| --- | --- | --- | --- | --- | --- | --- |
| For 2-hr sensitization Ca^2+^ studies | | | | | | |
| Donor 1 | 31 | F | 18.7 | Hispanic | Anoxia | BAM8-22 |
| Donor 2 | 18 | F | 28 | African American | Anoxia | BAM8-22, Histamine |
| Donor 3 | 26 | M | 26.3 | Hispanic | Head Trauma/GSW | BAM8-22, Histamine |
| Donor 4 | 43 | F | 27.4 | Caucasian | Head Trauma/Blunt injury | BAM8-22, Histamine |
| Donor 5 | 25 | M | 26.5 | Hispanic | Head Trauma/GSW | BAM8-22, Histamine |
| Donor 6 | 46 | M | 28.3 | Hispanic | Head Trauma/Blunt injury | AITC |
| Donor 7 | 50 | F | 20.1 | Asian | Anoxia/Drowning | AITC |
| Donor 8 | 21 | F | 27.3 | Caucasian | Head Trauma/Blunt injury | AITC, Capsaicin, |
| Donor 9 | 40 | M | 27.4 | African American | Head Trauma/GSW | Capsaicin |
| Donor 10 | 30 | M | 27.3 | Hispanic | Head Trauma/Blunt injury | Capsaicin |
| For 24-hr gene expression study (RNA seq. data) | | | | | | |
| Donor 11 | 49 | M | 32.3 | Hispanic | CVA/ICH/Stroke | - |
| Donor 12 | 39 | F | 27.9 | Caucasian | Anoxia/  Cardiovascular | - |
| Donor 13 | 30 | M | 25.8 | African American | Anoxia/Drug intoxication | - |
| For direct cytokine stimulation | | | | | | |
| Donor 14 | 35 | F | 17.9 | Caucasian | Anoxia | IL-4, IL-13, IL-33 |
| Donor 15 | 51 | M | 20.3 | Caucasian | CVA/ICH/Stroke | IL-4, IL-13, IL-33 |
| Donor 16 | 49 | M | 32.3 | Hispanic | CVA/ICH/Stroke | IL-4, IL-13, IL-33 |

AITC, allyl isothiocyanate; BMI, body mass index; CIS, control inflammatory stimuli; CVA, cerebrovascular accident; GSW, gunshot wound; ICH, intracerebral hemorrhage; IL, interleukin.

N = 5 donors

**Supplementary Table 2**. Results of differential gene expression analysis of human DRG cultures.

(Attached along with this manuscript)
