# Supplementary figures and images for: Type 2 cytokines sensitize human sensory neurons to itch-associated stimuli
Source: Front Mol Neurosci. 2023 Oct 5;16:1258823. doi: 10.3389/fnmol.2023.1258823 (PMC10586051; doi:10.3389/fnmol.2023.1258823)

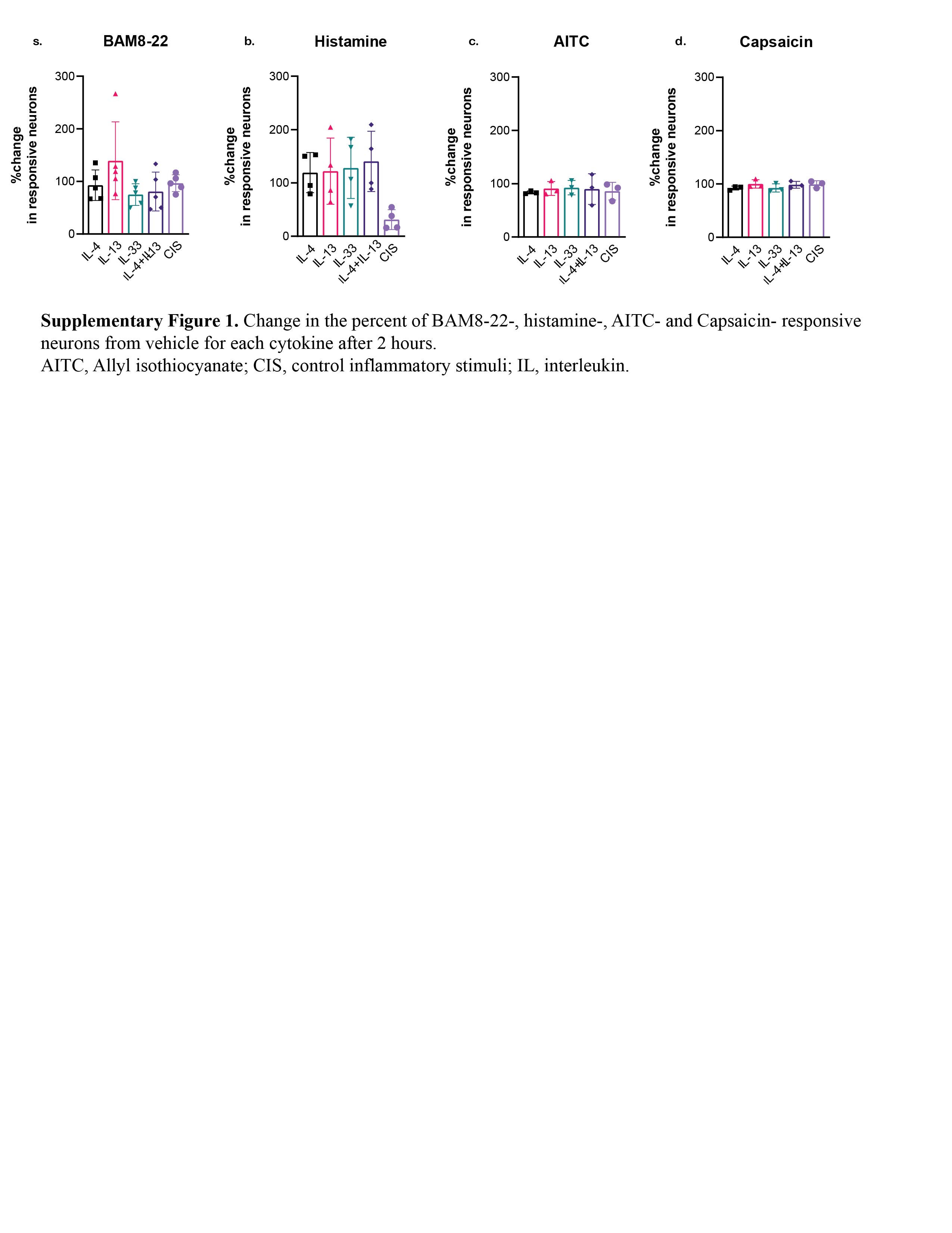

Supplement: Supplementary file 3 [file Image_1.jpg]
